# An Apparatus for Drainage

**Published:** 1897-07

**Authors:** M. L. Rhein

**Affiliations:** New York.


ARTICLE VII.

### AN APPARATUS FOR DRAINAGE.

BY DR. M. L. RHEIN, NEW YORK.

About four years ago there came under my hands for treatment a young lady, for whom I did considerable work before her mouth was placed in complete order. Previous to coming under my care she had considerable dental work done in Paris, and I found several teeth devitalized, the pulps not having been removed. About the end of the root of the right upper second bicuspid there was an abscess discharging freely. After particular care having been taken to thoroughly fill the root of this bicuspid to the very end, I informed her that it would be impossible to make a radical cure of this abscess without amputating the end of the root, and that if this were not done I would prefer the removal of the tooth rather than have it remain in position, especially taking into consideration its close proximity to the antrum.

She never gave me the opportunity of operating on this root. In fact, for two years I lost sight of the patient till about four or five weeks ago, when I was hastily called in consultation by her physician. She had been married in the interim, and was then pregnant about five months. When I saw her, there was a temperature of over 102° Fah., with a very considerable swelling in the region about the right eye. Referring to an old diagram of her mouth, showing exactly what I had done, I was soon able to diagnose a condition of antral abscess. I noticed that the bicuspid had been left by me with a large contour gold filling extending around both proximal sides of the tooth. I now saw that an amalgam filling had been inserted in the center of the crown. She vigorously denied, at first, that she had been under any other dental treatment since I had seen her last, but after pointing out to her the convincing proof shown by the amalgam filling, she conferred, with a little chagrin, that on feeling some irritation from the old abscess, she had consulted a dentist who had been recommended to her especially for the cheapness of his fee.

The tooth was extracted, and this was followed by a profuse discharge of pus. The opening into the antrum was small, taking only a fine probe. The dentist to whom she applied, instead of endeavoring to discover what was the condition of the interior of the root-canal, drilled through the center of the crown into the canal, striking there the oxiphosphate, covering the gutta-percha which sealed the canal. Endeavoring to drill through this, he proceeded but an eighth of an inch before he penetrated the side of the root, and then completed his operation by forcing a mass of pink gutta-percha fully half an inch through this artifical opening. The result of this increased irritation was a weakening of the floor of the antrum at this point, and the subsequent penetration of purulent germs into that cavity. My original filling can be seen reaching to the very end of the root.

Having on my hands a patient pregnant about five months, with a condition of chronic empyema, it was impossible to perform the surgical operation necessary for a cure of this condition, yet it was necessary to keep the antrum aseptic and clean. Syringing out the antrum through the fistula was painful.

As a substitute for syringing my associate, Dr. C. L. Andrews, materially assisted me in devising and manufacturing a little apparatus which I successfully inserted in her mouth, and by means of which the antrum was thoroughly cleansed without any inconvenience or annoyance, and by means of which she will be able to take the proper care of it daily for any length of time to come.

The apparatus is a curved plantinum tube, which fits perfectly into the alveolar socket, and is made a little larger after entering the antrum, so that the tissues will readily hold it in position, and it cannot easily be withdrawn with the apparatus that prevents the oral secretions from entering the tube. At the same time, the open ends of the tube at the gingival border flare over both the buccal and the palatal side of the process, so that it cannot be pushed up into the antrum.

In this tube is fitted an ordinary canula, with a knob soldered around it at the point where it should cease to enter the tube. To this canula is attached rubber tubing, with a stop-cock close to the canula. The tubing is attached to a glass jar suspended in a metal frame. By placing the cleansing solution in this jar and elevating it to the proper height, we could painlessly wash the antrum in the most thorough manner, the fluids leaving by way of the right nostril. This is done so easily that the patient will be able to cleanse the antrum daily as frequently as may be desired. To the open end of this tube has been accurately fitted a platinum cone-shaped nipple, which acts as a positive stopper to the tube. This nipple forms part of the under surface of a strip of platinum, to which is attached a porcelain tooth, and this in turn is held in perfect juxtaposition by means of clasps around the molar and first bicuspid, and with metal rests attached alongside of these clasps, and holding on to the occlusal points of the molar and bicuspid, so as to prevent the apparatus pressing upward on the gum.

The patient will use a cleansing solution of boracic acic and glycerol, and if the thorough cleansing by this method does not bring about a positive cure, a thorough surgical operation will be made.—*Dental Cosmos.*

